# Early Hemodynamic Profile after Aortic Valve Replacement - A Comparison between Three Mechanical Valves

**DOI:** 10.21470/1678-9741-2020-0273

**Published:** 2021

**Authors:** Khaled D. Algarni, Essam Hassan, Amr A. Arafat, Mostafa A. Shalaby, Hussein H. Elawad, Claudio Pragliola, Turki B. Albacker

**Affiliations:** 1 Department of Cardiac Sciences, College of Medicine, King Saud University, Riyadh, Saudi Arabia.; 2 Department of Adult Cardiac Surgery, Prince Sultan Cardiac Center, Riyadh, Saudi Arabia.; 3 Cardiothoracic Surgery Department, Tanta University, Tanta, Egypt.; 4 Adult Cardiology Department, Prince Sultan Cardiac Center, Riyadh, Saudi Arabia.

**Keywords:** Heart Valve Prosthesis, Aorta Valve, Isotonic Solutions, Bicarbonated Ringer's solutuin, Body Surface Area, Patient Discharge, Echocardiography

## Abstract

**Introduction:**

There are scarce data comparing different mechanical valves in the aortic position. The objective of this study was to compare the early hemodynamic changes after aortic valve replacement between ATS, Bicarbon, and On-X mechanical valves.

**Methods:**

We included 99 patients who underwent aortic valve replacement with mechanical valves between 2017 and 2019. Three types of mechanical valves were used, On-X valve (n=45), ATS AP360 (n=32), and Bicarbon (n=22). The mean prosthetic valve gradient was measured postoperatively and after six months.

**Results:**

Preoperative data were comparable between groups, and there were no differences in preoperative echocardiographic data. Pre-discharge echocardiography showed no difference between groups in the ejection fraction (*P*=0.748), end-systolic (*P*=0.764) and end-diastolic (*P*=0.723) diameters, left ventricular mass index (*P*=0.348), aortic prosthetic mean pressure gradient (*P*=0.454), and indexed aortic prosthetic orifice area (*P*=0.576). There was no difference in the postoperative aortic prosthetic mean pressure gradient between groups when stratified by valve size. The changes in the aortic prosthetic mean pressure gradient of the intraoperative period, at pre-discharge, and at six months were comparable between the three prostheses (*P*=0.08). Multivariable regression analysis revealed that female gender (beta coefficient -0.242, *P*=0.027), body surface area (beta coefficient 0.334, *P*<0.001), and aortic prosthetic size (beta coefficient -0.547, *P*<0.001), but not the prosthesis type, were independent predictors of postoperative aortic prosthetic mean pressure gradient.

**Conclusion:**

The three bileaflet mechanical aortic prostheses (On-X, Bicarbon, and ATS) provide satisfactory early hemodynamics, which are comparable between the three valve types and among different valve sizes.

**Table:** 

Abbreviations, acronyms & symbols				
AR	= Aortic regurgitation		EuroSCORE	= European System for Cardiac Operative Risk Evaluation
AS	= Aortic stenosis		LV	= Left ventricular
AVR	= Aortic valve replacement		MPG	= Mean pressure gradient
BSA	= Body surface area		MV	= Mitral valve
CABG	= Coronary artery bypass grafting		MVR	= Mitral valve replacement
CI	= Confidence interval		TEE	= Transesophageal echocardiography
CPB	= Cardiopulmonary bypass		TTE	= Transthoracic echocardiography
EDD	= End-diastolic diameter		TV	= Tricuspid valve
EOA	= Effective orifice area		TVR	= Tricuspid valve replacement
ESD	= End-systolic diameter		VIF	= Variance inflation factor

## INTRODUCTION

Over the past decades, different mechanical prosthetic valves have been innovated and refined to be used in both aortic and mitral positions. The choice of optimal valve has been based on the results of numerous studies, regional differences, and the anti-thrombotic treatments available^[1]^. Besides durability, there are numerous hemodynamic parameters such as pressure gradients, effective orifice area, and energy loss, which dictate the choice for a specific valve^[1]^. We are now in the era of bileaflet mechanical valves, which have been used for more than thirty years and been proved to provide superior hemodynamics, durability, and low thrombogenicity^[2]^.

The current American and European guidelines for the management of valvular heart disease state that mechanical valve prosthesis remains the 'standard of care' for patients less than 60 years of age who do not have a contraindication to anticoagulation^[3,4]^.

The ideal prosthetic valve is yet to be developed, and several studies showed comparable outcomes among the new generation of mechanical valves^[5,6]^. Recently, we started to increasingly use the On-X aortic prosthesis due to the advantage of lower anticoagulation targets. The objective of this study was to compare the early hemodynamic performance of the three aortic prostheses that we currently use (On-X, ATS AP360, and Bicarbon).

## METHODS

This is a retrospective study that was conducted on patients who received a mechanical aortic valve as a part of surgical aortic valve replacement (AVR), either isolated or combined with other cardiac procedures. The study was conducted during the period between January 2017 and December 2019. The rationale for using this time period is the fact that we started using On-X aortic prosthesis in early 2017. Data collection was approved by the Institutional Review Board, and patients' consent was waived.

During the study period, 271 patients underwent AVR. Patients who received bioprosthetic aortic valves, underwent aortic root replacement with a composite graft, and one patient who had a replacement with St. Jude mechanical valve were excluded. Ninety-nine patients who received mechanical AVR were included in this study.

Three types of mechanical prostheses were used in this study: On-X bileaflet mechanical valve (On-X Life Technologies, Austin, Texas, United States of America) in 45 patients, ATS AP360 (Medtronic Medical, Santa Rosa, California, United States of America) in 32 patients, and Bicarbon (LivaNova PLC, Sorin, Sluggia, Italy) in 22 patients. The choice of the mechanical valve type was based on the primary surgeon's preference.

Most procedures (n=93) were performed through a median sternotomy, and six patients had a minimally invasive AVR through upper J-sternotomy. Cardiopulmonary bypass (CPB) and cardioplegia techniques were also influenced by the surgeon. Either normothermic CPB with intermittent warm blood cardioplegia for myocardial protection or moderate hypothermic CPB with single-dose Del Nido blood cardioplegia was used. All valves were implanted in the supra-annular position. Transesophageal echocardiography (TEE) was routinely performed before surgery and after separation from CPB. Most patients' demographic, intraoperative, and postoperative data were prospectively collected in a computerized database. Variables that were not collected in the prospective database were collected retrospectively from electronic records and then added to the database. Pre-discharge transthoracic echocardiography (TTE) was done for all cases.

### Echocardiography

Preoperative transthoracic echocardiography (TTE) was routinely performed in all patients to evaluate the valve and cardiac functions. All patients had TEE performed intraoperatively before commencing and after completion of surgery. All patients had TTE evaluation postoperatively before hospital discharge. Likewise, all patients had a follow-up TTE study a few months after surgery. TTE at a six-month follow-up was available for the majority of patients (n=93, 93.9%). In patients who had less than a six-month follow-up (n=6, 6.1%), we relied on the TTE performed at a three-month follow-up after surgery. For the purpose of the study, one experienced echocardiographer reviewed all the echocardiographic measurements. The echocardiographic evaluation was conducted according to the American Society of Echocardiography guidelines^[7]^. The mean aortic prosthetic pressure gradient was calculated from the area under the curve of the systolic trans-prosthetic flow spectrum. Echocardiographic follow-up data were 99% complete.

### Statistical Analysis

Continuous variables were presented as means with standard deviation or medians with 25^th^ and 75^th^ percentiles for variables with non-normal distribution. All continuous variables were explored for normality of distribution using the following normality diagnostics: histograms and skewness. Continuous variables were compared using a one-way analysis of variance test if normally distributed and Kruskal-Wallis test for variables with non-normal distribution. Categorical variables were presented as numbers and percentages and compared using Chi-square or Fisher's exact test if the expected frequency is < 5.

A repeated measures analysis was used for the analysis of mean aortic pressure gradient to test the main effect of time (preoperative, intraoperative, pre-discharge, and six months), valve type (ATS, On-X, and Bicarbon), and the time × valve type interaction to determine the change in aortic prosthetic mean pressure gradient over the four-time points by valve type. The linear regression model was used to evaluate independent predictors of the postoperative mean aortic pressure gradient. The univariate linear regression analysis was performed to screen potential confounders (independent variables) of the postoperative aortic mean pressure gradient (dependent variable). The following variables were screened with univariate linear regression analysis: age, gender, body surface area (BSA), valve type (dummy variable), prosthesis size (as a continuous variable), aortic regurgitation (AR), aortic stenosis (AS), concomitant mitral valve replacement (MVR), concomitant coronary artery bypass grafting, reoperation, left ventricular ejection fraction, and preoperative aortic valve mean gradient. Variables that had a *P*-value <.2 on the univariate analysis (gender, BSA, prosthesis size, AR, AS, concomitant MVR, prosthesis size) and variables that are considered clinically important (age, valve type as dummy variables, and preoperative aortic mean pressure gradient) were submitted to a multivariable linear regression model using the backward elimination method with a *P*-value entry and removal criteria of .05 and .1, respectively. The model was evaluated for multicollinearity using the variance inflation factor (VIF). We used a conservative threshold (VIF=2.5) to diagnose collinearity. IBM Corp. Released 2019, IBM SPSS Statistics for Windows, Version 26.0, Armonk, NY: IBM Corp. was used to perform the statistical analysis. A two-sided *P*-value < 0.05 was considered statistically significant.

## RESULTS

### Baseline and Operative Data

The age was comparable between groups. On-X valves were implanted more in males compared to Bicarbon and ATS valves. Other preoperative variables were comparable between groups; Table 1 details the preoperative patients' characteristics, concomitant procedures, and valve sizes. Aortic annular enlargement was performed for one patient using the Nicks technique. Approximately 22% (n=22) of patients had small aortic prosthesis; 3% of patients (n=3) had size 16 mm aortic prosthesis and 19% (n=19) had size 18 mm prosthesis. However, we have to point out that 16% (n=16) of the patients had a BSA of 1.6 m^2^ or less, and 10% (n=10) had a BSA of 1.5 m^2^ or less. The 5^th^, 10^th^, and 25^th^ percentiles for the BSA of the population of this study are 1.4 m^2^, 1.5 m^2^, and 1.7 m^2^, respectively. So, 25% of patients in this study have a BSA of 1.7 m^2^ or less.

**Table 1:** Baseline characteristics and operative data.

Variable	ATS (n= 32)	Bicarbon (n= 22)	On-X (n= 45)	*P*-value
Age (years)	46.44±12.16	41.82±12.01	40.89±12.38	0.14
Male	11 (34.4%)	13 (59.1%)	34 (75.6%)	0.001
BSA	1.83±0.20	1.82±0.24	1.87±0.22	0.58
EuroSCORE II	4.95±7.86	3.82±4.49	2.81±2.71	0.22
AR	16 (50%)	13 (59.09%)	21 (46.67%)	0.63
AS	9 (28.13%)	3 (13.64%)	7 (15.56%)	0.31
Combined AS+AR	7 (21.88%)	6 (27.27%)	17 (37.78%)	0.31
Reoperative AVR	8 (25.0%)	6 (27.3%)	13 (28.9%%)	0.93
Concomitant CABG	2 (6.3%)	2 (9.1%)	2 (4.4%)	0.86
Concomitant MV repair	0	1 (4.5%)	2 (4.4%)	0.45
Concomitant MVR	20 (62.5%)	10 (45.45%)	16 (35.56%)	0.07
Concomitant ascending aorta replacement	2 (6.3%)	1 (4.5%)	2 (4.4%)	0.90
Concomitant TV repair	7 (21.9%)	6 (27.3%)	6 (13.3%)	0.43
Concomitant TVR	2 (6.3%)	2 (9.1%)	2 (4.4%)	0.86
Aortic valve size (mm)				< 0.001
16	1 (3.1%)	2 (9.1%)	-	
18	10 (31.3%)	9 (40.9%)	-	
19	-	-	9 (20.0%)	
20	10 (31.3)	5 (33.3%)	-	
21	-	-	16 (35.6%)	
22	7 (21.9%)	4 (18.2%)	-	
23	-	-	15 (33.3%)	
24	4 (12.5%)	2 (9.1%)	-	
25	-	-	3 (6.7%)	
27	-	-	2 (4.4%)	
Total	32 (100%)	22 (100%)	45 (100%)	

AR=aortic regurgitation; AS=aortic stenosis; AVR=aortic valve replacement; BSA=body surface area; CABG=coronary artery bypass grafting; EuroSCORE=European System for Cardiac Operative Risk Evaluation; MV=mitral valve; MVR=mitral valve replacement; TV=tricuspid valve; TVR=tricuspid valve replacement

### Preoperative Echocardiographic Data

There were no differences between groups in the preoperative left ventricular ejection fraction, end-systolic diameter, left ventricular mass index, and aortic valve mean pressure gradient (Table 2).

**Table 2:** Preoperative echocardiographic data.

Variable	ATS (n= 32)	Bicarbon (n= 22)	On-X (n= 45)	*P*-value
Ejection fraction (%)	51.09±8.77	51.14±11.33	52.22±9.97	0.86
EDD (cm)	5.18±0.94	5.13±0.90	5.70±1.04	0.03
ESD (cm)	3.46±1.03	3.63±0.94	3.91±0.96	0.13
LV mass index (g/m^2^)	113.31±39.33	128.03±29.88	130.27±44.92	0.18
Aortic valve MPG (mmHg)	35.07±20.91	35.11±21.29	37.12±25.72	0.92
Peak aortic jet velocity (Vmax cm/s)	352.77±126.15	353.03±146.61	403.72±321.46	0.59

EDD=end-diastolic diameter; ESD=end-systolic diameter; LV=left ventricular; MPG=mean pressure gradient

### Postoperative Outcomes

Pre-discharge echocardiography showed no differences between groups in the postoperative left ventricular ejection fraction, end-systolic and end-diastolic diameters, left ventricular mass index, mean aortic pressure gradient, and indexed aortic orifice area (Table 3).

**Table 3:** Postoperative echocardiographic data.

Variable	ATS (n= 32)	Bicarbon (n= 22)	On-X (n= 45)	*P*-value
Ejection fraction (%)	48.91±10.76	47.14±12.71	49.41±10.72	0.75
EDD (cm)	4.94±0.99	5.10±1.09	4.90±0.70	0.72
ESD (cm)	3.66±1.13	3.74±1.35	3.54±0.75	0.76
LV mass index (g/m^2^)	109.01±41.34	105.97±42.46	123.46±55.89	0.35
Aortic prosthetic MPG (mmHg)	14.6 ±6.3	17.3±4.8	15.8±6.3	0.45
Aortic valve velocity (Vmax cm/s)	253.69±57.12	274.45±40.21	256.67±60.85	0.36
Indexed EOA (cm/m^2^)	1.83±0.20	1.82±0.24	1.87±0.22	0.58

EDD=end-diastolic diameter; EOA=effective orifice area; ESD=end-systolic diameter; LV=left ventricular; MPG=mean pressure gradient

**Fig. 1:**
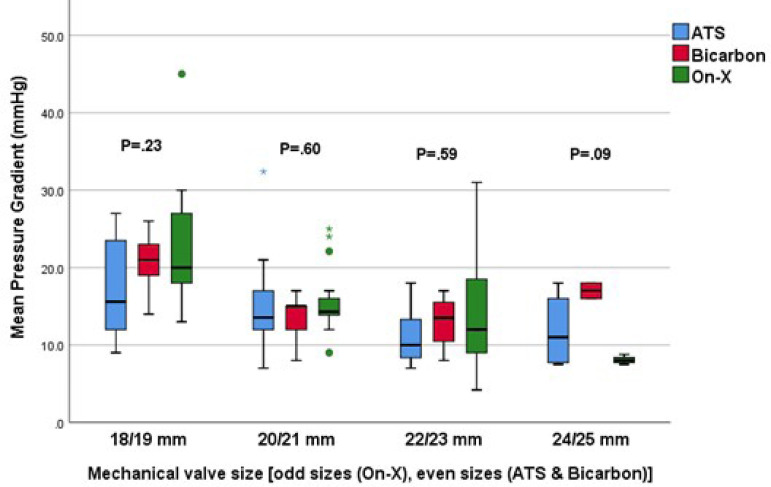
The mean pressure gradient of the aortic prostheses postoperatively based on pre-discharge transthoracic echocardiography (TTE) for different valve sizes (18/19 mm, 20/21 mm, 22/23 mm, and 24/25 mm). Size 16 and size 26/27 are not shown in the graph due to small numbers. Even number sizes are for Bicarbon and ATS AP360 valves, while odd number sizes are for On-X. The grouping of the On-X with the Bicarbon and ATS AP360 was based on the nearest external diameter.

There were no differences in the postoperative aortic valve mean pressure gradients between the three valve types when stratified by valve size (Figure 1). The changes in the mean aortic valve pressure gradient of the intraoperative period, at pre-discharge, and after six months were comparable between the three valve types (*P*=0.08). Although there was a trend of higher aortic mean pressure gradient with Bicarbon aortic valve, this difference did not reach a statistical significance (*P*=0.08). Moreover, the use of small sizes (16 mm and 18 mm) was more frequent with Bicarbon prosthesis, which may explain the nonsignificant trend of the higher mean pressure gradient in the Bicarbon valve group. The change in aortic prosthetic pressure gradient over the four-time points was significant across all prosthetics types where the mean gradient decreased significantly after valve replacement and then increased significantly from the intraoperative period to pre-discharge and then at six months (*P*<0.001) (Table 4). The small but statistically significant increase in the aortic mean pressure gradient at discharge and at the six-month follow-up TTE compared to intraoperative TEE is likely related to changes in hemodynamics and loading conditions.

**Table 4:** Aortic mean pressure gradient (mmHg) at four-time points.

Valve type	Preoperative TEE	Postoperative TEE	Pre-discharge TTE	6-month TTE	*P*-value*
	MPG (time 1)	MPG (time 2)	MPG (time 3)	MPG (time 4)	
ATS	35.1±20.9	11.4±6.4	14.6±6.3	17.04±8.4	< 0.001
Bicarbon	35.1±21.3	13.7±6.2	17.3±4.8	21.5±12.9	< 0.001
On-X	37.1±25.7	12.2±4.4	15.8±6.3	17.3±7.6	< 0.001

MPG=mean pressure gradient; TEE=transesophageal echocardiography; TTE=transthoracic echocardiography

**Fig. 2:**
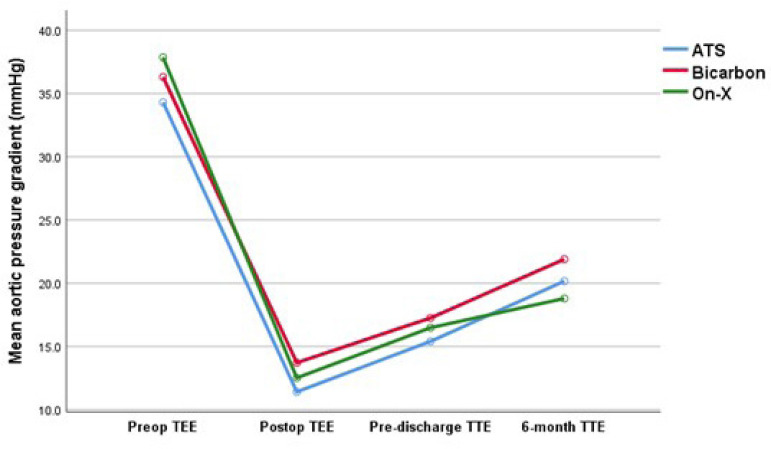
Estimated marginal means of aortic valve mean pressure gradients (MPG) at four-time points (preoperative transesophageal echocardiography [TEE], postoperative TEE, pre-discharge transthoracic echocardiography [TEE], and six-month follow-up TTE) for the three valve types (ATS, On-X, and Bicarbon) adjusted for valve sizes. Repeated measure analysis did not show a significant difference in MPG between the three aortic valve types over the four-time points (Wilks' Lamda P=0.08). However, the change in MPG across the four-time points was significant for all three valves (Wilks' Lambda P<0.001, Partial Eta Squared=0.295). Pairwise comparisons between the four-time points are all significant (P<0.001 for time 1 vs. time 2, time 3, and time 4; P<0.001 for time 2 vs. time 3; and P=0.006 for time 3 vs. time 4). Estimated marginal means with the 95% confidence interval for the four-time points, respectively, for the three valve prostheses are as follow: ATS 34.3 (26.5-42.2), 11.4 (9.4-13.4), 15.4 (13-17.8), and 20.2 (16.8-23.5); Bicarbon 36.3 (27-45.9), 13.7(11.3-16.2), 17.3 (14.4-20.1), and 21.9 (17.8-26); and On-X 37.9 (31.1-44.6), 12.5 (10.8-14.2), 16.5 (14.5-18.5), and 18.8 (15.9-21.7).

* *P*-value is based on repeated measures analysis. Repeated measures analysis did not show a significant difference in MPG between the three aortic valve types over the four-time points (Wilks' Lamda *P*=0.08). However, the change in MPG across the four-time points was significant for all three valves (Wilks' Lambda *P*<0.001, Partial Eta Squared=0.295). Pairwise comparison between the time points are all significant (*P*<0.001 for time 1 *vs*. time 2, time 3, and time 4; *P*<0.001 for time 2 *vs*. time 3; and *P*=0.006 for time 3 *vs*. time 4). The estimated marginal means adjusted for valve sizes are depicted in Figure 2.

### Independent Predictors of Postoperative Aortic Prosthetic Mean Pressure Gradient

Multivariable linear regression analysis revealed that the female gender (coefficient -0.242, *P*=0.027), BSA (coefficient 0.334, *P*<0.001), and aortic valve size (coefficient -0.547, *P*<0.001) were independent predictors of postoperative prosthetic aortic valve mean gradient. Valve type was not a predictor of aortic prosthetic mean gradient (Table 5).

**Table 5:** Multivariable linear regression of factors affecting the postoperative prosthetic aortic valve mean pressure gradient.

Variable	B	95% CI of B	β	*P*-value
Constant	26.4	13.6-39.3	-	< 0.001
Gender*	-2.8	5.4- -0.33	-0.24	0.027
BSA	8.9	4.2-13.6	0.33	< 0.001
Aortic valve size	-1.4	-1.8- -0.84	-0.54	< 0.001

BSA=body surface area; CI=confidence interval

* Male is the reference group

The following variables were included in the multivariable linear regression model using the backward elimination method (age, gender, BSA, valve type, prosthesis size [as a continuous variable], aortic regurgitation, aortic stenosis, concomitant mitral valve replacement, and preoperative aortic valve mean gradient). Model R (.55); R Square (.31) and adjusted R square (.28). The model was checked for multicollinearity with the variance inflation factor (VIF). All values for VIF were < 2.

## DISCUSSION

In this retrospective study, including 99 patients who were operated during the same time periods between 2017 and 2019, we compared early hemodynamics of three different types of commercially available bileaflet mechanical aortic prosthesis (On-X, ATS, and Bicarbon). All three valves provided satisfactory and comparable early hemodynamics. The mean pressure gradient for the three valves was comparable over time up to six months. There was a trend of higher mean pressure gradient with the Bicarbon valve; however, that did not reach a statistical significance. Moreover, this nonsignificant difference is likely explained by the more prevalent small sizes (sizes 16 and 18) in the Bicarbon group compared to other groups. Indeed, on multivariable linear regression analysis with adjustment for valve sizes, the prosthesis type did not influence the mean pressure gradient.

This is the first study that compared these three mechanical valves head to head, but other studies have shown similar results with other valid comparisons. Walther et al. compared On-X and St. Jude HP aortic valves in 40 patients and found no difference between groups regarding the maximum pressure gradient, both postoperatively and after a one-year follow-up^[8]^. Xu et al. compared the early hemodynamics after AVR with On-X and St. Jude prostheses. Each group had 33 patients, and they found no difference in postoperative peak gradient across the prosthesis in both groups. However, for the valve size 25 mm, On-X valves had a lower peak gradient but no difference in the effective orifice area^[5]^. We found a similar finding where there was a nonsignificant trend towards lower mean pressure gradient in the 25 mm On-X valve size compared to 24 mm ATS and Bicarbon valve sizes (*P*=0.09), as depicted in Figure 2.

Valve type was not an independent predictor of aortic prosthetic mean pressure gradient after adjustment for confounders. We found that BSA, female gender, and valve size are independently associated with the postoperative aortic prosthetic mean pressure gradient. Similarly, Tully et al. found female sex and BSA as independent determinants of postoperative indexed effective orifice area. They also found that female patients and patients with small BSA had more prevalence of patient-prosthesis mismatch^[9]^.

Approximately one third (31.1%) of our patients in the present study received small aortic prosthesis (size 19 or smaller), and since those patients had a smaller BSA compared to patients who received larger prosthesis, we had satisfactory results with small sizes' valves with a mean gradient of 14.5±6.2 *vs*. 11.5±5.4 mmHg in the small prosthesis group (prosthesis size 19 mm or smaller) and the large prosthesis group (prosthesis size > 19 mm), respectively (*P*=0.01). Kato et al. examined short and mid-term outcomes on 78 patients who underwent AVR with ATS prosthesis size 16-18 mm and SJM Regent size 17 mm. They found that the postoperative pressure gradient of the ATS AP 16 mm group was significantly higher than that of the ATS AP 18 mm group; however, the left ventricular mass decreased substantially during the follow-up in all groups, and the extent of reduction in left ventricular mass was similar among the groups (-30%, -25%, and -28% in the 16 mm ATS AP, 17 mm Regent, and 18 mm ATS AP groups, respectively; *P*=0.844). They concluded that standard AVR using these small mechanical valves, which avoids the need to enlarge the annulus or to conduct more complex stentless bioprosthesis implantation, might represent an acceptable solution, especially in elderly patients with a small aortic root^[10]^.

In our study, there was no valve-related bleeding or thrombosis up to six months for all three prosthesis types. Since all three valves have similar early hemodynamic profiles, we see the advantage of the On-X valve in terms of lower anticoagulation target International Normalised Ratio (1.5-2), which can reduce the incidence of bleeding complications. Indeed, in a recent multicenter, long-term study by Chambers et al. including 691 patients who had an AVR and MVR using On-X valves, the late rate (mean of 5.2 years) of aortic valve thromboembolism, bleeding, and thrombosis were 0.6%, 0.4%, and 0%, respectively^[11]^. Nevertheless, we also see the benefits of other mechanical valves (ATS AP 360 and Bicarbon) especially in patients with small BSA and small aortic annulus as the smaller size for On-X aortic valve is 19 mm, while both ATS and Bicarbon valves are available in smaller sizes (16 mm and 18 mm). It is important, however, to determine the indexed effective orifice area before deciding to implant these small aortic prostheses to avoid significant patient-prosthesis mismatch.

### Limitations

This is a retrospective single-center study with the inherent limitations of this study design, including selection and information bias. Another limitation is the small sample size. Additionally, the subgroup analysis by the valve size category is limited by the small sample size and is not powered to detect potential significant differences, as it is evident by the wide 95% confidence intervals. For future studies, we estimated that a sample size of approximately 471 patients is required for adequately powered subgroup analyses to compare the mean aortic pressure gradient of various valve sizes. Furthermore, many variables that may affect the outcomes may be unequally distributed between groups. However, we performed a multivariable regression analysis to adjust for these confounders that may have affected the postoperative mean prosthetic valve gradient. Finally, we only reported early (six-month) outcomes, and therefore, we cannot make comparisons about long-term outcomes between these three mechanical valves.

## CONCLUSION

In summary, bileaflet mechanical aortic valves, On-X, Bicarbon, and ATS AP360 provide satisfactory early hemodynamics. The hemodynamics were comparable between the three valve types and among different valve sizes. Hemodynamics for small valve sizes (16-19 mm) were satisfactory for all three valves.

**Table:** 

**Authors' roles & responsibilities**	
KDA	Substantial contributions to the design of the work; analysis of data for the work; drafting the work and revising it; final approval of the version to be published
EH	Acquisition of data for the work; drafting the work and revising it; final approval of the version to be published
AA	Substantial contributions to the design of the work; revising the draft of the work; writing the final manuscript; final approval of the version to be published
MAS	Substantial contributions to the design of the work, acquisition of data for the work; revising the draft of the work; final approval of the version to be published
HHE	Acquisition of data for the work; drafting the work and revising it; final approval of the version to be published
CP	Substantial contributions to the conception and design of the work; revising the draft of the work; final approval of the version to be published
TBA	Substantial contributions to the conception of the work; supervision; drafting the work and revising it; final approval of the version to be published
